# Measurement properties and responsiveness of the EQ-5D-Y-5L compared to the EQ-5D-Y-3L in children and adolescents receiving acute orthopaedic care

**DOI:** 10.1186/s12955-022-01938-6

**Published:** 2022-02-17

**Authors:** Janine Verstraete, Zara Marthinus, Stewart Dix-Peek, Des Scott

**Affiliations:** 1 Department of Paediatrics and Child Health, Division of Pulmonology, Cape Town, South Africa; 2 Department of Paediatrics and Child Health, Orthopaedic Surgery, Cape Town, South Africa; 3 Division of Physiotherapy, Maitland Cottage Hospital, Cape Town, South Africa; 4 Faculty of Health and Rehabilitation Sciences, Division of Physiotherapy, Cape Town, South Africa

**Keywords:** Orthopaedic, Fracture, Surgery, Children, Adolescents, Youth, Health related quality of life, EQ-5D-Y, Three level, Five level, EQ-5D-Y-5L

## Abstract

**Objective:**

The aim of this study is a head-to-head comparison of the instrument performance and responsiveness of the EQ-5D-Y-3L and the expanded English version of the EQ-5D-Y-5L in children/adolescents receiving acute orthopaedic management in South Africa.

**Methods:**

Children/adolescents aged 8–15 years completed the EQ-5D-Y-5L, EQ-5D-Y-3L, self-rated health (SRH) question and PedsQL at baseline. The EQ-5D-Y-5L, EQ-5D-Y-3L and SRH question were repeated after 24 and 48 h. Performance of the EQ-5D-Y-5L and EQ-5D-Y-3L was determined by comparing feasibility (missing responses), redistribution of dimensions responses, discriminatory power, concurrent validity, and responsiveness.

**Results:**

Eighty-three children/adolescents completed baseline measures and seventy-one at all three time-points. Reporting of 11111 decreased by 20% from the EQ-5D-Y-3L to the EQ-5D-Y-5L. Informativity of dimensions improved on average by 0.267 on the EQ-5D-Y-5L with similar evenness. There was a range of 11–27% inconsistent responses when moving from the EQ-5D-Y-3L to the EQ-5D-Y-5L. There was a low to moderate and significant association on the EQ-5D-Y-3L and EQ-5D-Y-5L to similar items on the PedsQL and SRH scores. Percentage change over time was greater for the EQ-5D-Y-5L (range 0–182%) than EQ-5D-Y-3L (range 0–100%) with the largest reduction for both measures between 0 and 48 h. For those who respondents who showed an improved SRH the EQ-5D-Y-5L and EQ-5D-Y-3L showed significant paired differences.

**Conclusion:**

The English version of the EQ-5D-Y-5L appears to be a valid and responsive extension of the EQ-5D-Y-3L for children receiving acute orthopaedic management. The expanded levels notably reduce the ceiling effect and has greater discriminatory power. Concurrent validity of the EQ-5D-Y-3L and EQ-5D-Y-5L was low to moderate with similar PedsQL items and SRH. The EQ-5D-Y-5L generally showed greater change than the EQ-5D-Y-3L across all dimensions with the greatest change observed for 0–48 h. Responsiveness was comparable across the EQ-5D-Y-3L and EQ-5D-Y-5L for those with improved SRH. Greater sensitivity to change may be observed on comparison of utility scores, once preference-based value sets are available for the EQ-5D-Y-5L.

## Background

The measurement of self-reported health in children and adolescents has been used increasingly in population health surveys, clinical trials and for studies of routine health care [1]. The EQ-5D-Y has been widely used to measure and value health in younger populations aged 8–15 years [2]. In the 18 years following its development it was reported to have been registered for use in 586 studies [1], which has likely increased as it is now available in over 50 language versions across multiple modes of completion. It is anticipated that the use of the measure in decision making will increase now that the first preference-based value sets have been published [3, 4].

The advantages of the EQ-5D-Y includes the simplicity of the descriptive system which measures health across five dimensions and a general rating of health on a visual analogue scale (VAS) of 0 (worst health) to 100 (best health) [2]. The dimensions include *Mobility* (walking about), *Looking After Myself* (washing and dressing), *doing Usual Activities* (going to school, hobbies, sports, playing, doing things with family or friends), *having Pain or Discomfort* and *feeling Worried, Sad or Unhappy*. The original youth version, EQ-5D-Y-3L, describes health on three levels (no problems, some problems and a lot of problems) which results in 243 (3^5^) health states [2, 5]. The three levels of report on this measure however seems to limit its sensitivity to measuring health and change in health across time. Thus, the response options of the youth version, EQ-5D-Y, were recently expanded to five levels [no/not, a little bit, some/quiet, a lot/really, cannot/extreme(ly)], resulting in 3125 (5^5^) health states [6]. Expanding the response option on the EQ-5D-Y-3L has generally shown improved performance in general population and patient populations with decreased ceiling effect when compared to the expanded five level version, EQ-5D-Y-5L[7–15].

A head-to-head comparison of responsiveness in paediatric patients with idiopathic scoliosis, aged 8–17 years, showed that the EQ-5D-Y-5L had comparable responsiveness to the EQ-5D-Y-3L [15]. The sample in this study had largely (82.7%) unchanged health over the study period thus decreasing the opportunity to determine responsiveness. Evaluation of outcome post orthopaedic management is becoming increasingly important considering the high burden on health care services with fractures alone accounting for 10% of children presenting to emergency medical services across Europe [16] with a higher incidence in South Africa [17]. The current recommendation following a literature review by Marson et al. [16] is that the EQ-5D-Y-3L and PedsQL should be used to evaluate Health Related Quality of Life in orthopaedic treatment. The aim of this study was thus to investigate the feasibility, redistribution and discriminatory power of dimension responses, concurrent validity, and responsiveness of the EQ-5D-Y-3L and the EQ-5D-Y-5L in children and adolescents receiving acute orthopaedic management.

## Methods

### Study design and participants

An observational, descriptive study with repeated measures for responsiveness was conducted. Children/adolescents requiring acute medical treatment for a traumatic or chronic congenital/acquired orthopaedic condition were recruited from the inpatient wards of an acute tertiary paediatric hospital and a specialist paediatric orthopaedic hospital. The majority of patients admitted to the facility have surgical intervention for paediatric orthopaedic conditions which often requires serial correction [18] or complex multi-level surgery [19, 20]. Those with traumatic fractures are managed with surgical correction and are admitted to the specialist orthopaedic hospital for rehabilitation before discharge. For those with fractures which are not amenable to surgery, are often managed on traction. Both medical facilities place a strong focus on physiotherapy with early rehabilitation and mobilisation with aim for early discharge. The average length of hospital stay is 7.8 days, children with complex chronic orthopaedic conditions staying longer than those with traumatic conditions.

All children/adolescents aged 8–15 years, who were able to read and write English, at each facility were eligible for the study. Only those who returned a signed informed consent and assent were included in the study and those who were medically unstable were excluded as the research may have been too distressing. All the children in the study requiring surgical management of their orthopaedic condition, completed the baseline questionnaires after surgery. Responsiveness of the measures was assessed with repeat measures after 24 and 48 h and it was anticipated that there would be a decrease in reporting of *having Pain or Discomfort* and problems with *Mobility* over this period of time before they were likely discharged home. It was further anticipated that the EQ-5D-Y-5L may be able to better discriminate between small changes in health state which are likely to occur in this patient group over short periods of time in the acute setting and before discharge home. Considering the clinical recovery of children post orthopaedic intervention it was anticipated that there would be change in their condition daily with the greatest change expected between baseline and 48 h, with discharge soon thereafter. Most children are expected to return to previous activities, including school, within a fortnight of surgery [21]. Due to the underlying chronic condition of some of those receiving paediatric orthopaedic management and the serial nature of correction, responsiveness was not measured over a longer period of time as many other long-standing factors may have affected their health state. As some of those receiving orthopaedic management did not have scheduled or elective orthopaedic management data was not collected before and after treatment.

### Instruments

#### EQ-5D-Y

The official self-report EQ-5D-Y-3L English version for South Africa was used in this study. The experimental EQ-5D-Y-5L English version for the United Kingdom was tested for equivalence in English for South Africa by the EuroQol group before it was used in this study. This EQ-5D-Y-5L version was further tested for interpretation of severity qualifiers with the rank order task as described by Derrett et al. (2021). The three or five levels of the descriptive system are expressed with a five-digit code. For example, the EQ-5D-Y-3L health state 11223 describes someone with no problems with *Mobility*, no problems with *Looking After Myself*, some problems with *doing Usual Activities*, having some *Pain or Discomfort* and feeling very *Worried, Sad or Unhappy*. The best health state described by the instrument is coded as 11111, describing ‘no problems’ in each of the dimensions [22]. Although the EQ-5D-Y-3L has a preference-based score the EQ-5D-Y-5L does not [3, 4]. As such a level sum score (LSS) was used to describe the responses on the descriptive system where the level labels are treated as numeric data with the best possible score (1 + 1 + 1 + 1 + 1) = 5 and the most severe score for the EQ-5D-Y-3L is (3 + 3 + 3 + 3 + 3) = 15. The other health states will have a LSS ranging between 5 and 15, with a larger score indicating a worse health state. EQ-5D-Y-5L is similarly scored with a LSS ranging between 5 and 25 [23]. This is a crude measure with limitations [24, 25] but gives some indication of the performance of the dimensions between the EQ-5D-Y-3L and EQ-5D-Y-5L. The adult value sets, EQ-5D-3L and EQ-5D-5L, were not considered suitable for the youth instruments, EQ-5D-Y-3L and EQ-5D-Y-5L, considering the differences in descriptor systems [3, 4]. Thus, the LSS is likely to give an indication of performance of the EQ-5D-Y-3L and EQ-5D-Y-5L..

##### Pediatric Quality of Life Inventory (PedsQL)

The 23 item PedsQL Generic Core Scales for children aged 8–12 years and 13–18 years were used as appropriate [26]. Both age versions of the PedsQL consist of self-reporting on four dimensions of functioning: physical, emotional, social, and school with 8, 5, 5 and 5 items respectively. Each item is scored on a Likert scale from 0 to 4 (never a problem to almost always a problem). Items are reversed scored and transformed to a 0–100 scale: 0 = 100, 1 = 75, 2 = 50, 3 = 25, 4 = 0. Dimension scores are calculated by a sum of the item scores divided by the total number of items. A total score is similarly generated by summing the dimension scores over the total number of dimensions giving an overall Health Related Quality of Life (HRQoL) score. Scores for scales with more than 50% missing data are not computed. A higher PedsQL score indicates a better HRQoL. The PedsQL is a profile measure which has been utilised previously to explore the concurrent validity of the EQ-5D-Y [7, 27–29].

##### Self-Rated Health (SRH)

The Self-Rated Health (SRH) question asks the child to describe their general health today as: ‘excellent’, ‘very good’, ‘good’, ‘fair’ or ‘poor’. This will allow sub-group analysis of children according to self-perceived general health and allow a yardstick against which to measure improvement of health in responsiveness testing. This question has been shown to be a valid measure of subjective health in children and adolescents [30]. Furthermore, it was used as an outcome measure to test the validity and reliability of the EQ-5D-Y-3L in a multi-national study [31]. The items were scored numerically for data analysis with excellent scored 5 and poor scored 1. For responsiveness testing if the score between two time points was identical it was considered unchanged (e.g. scored good health at 24 and 48 h). If the score between time points was different it was classified as either improved (e.g. if the score changed from poor to fair/good/very good/excellent) or worsened (e.g. if the score changed from very good to good/fair/poor). This does not capture the magnitude of change but rather any change in self-rated health.

## Procedure

Ethics approval was obtained from the University of Cape Town, Faculty of Health Sciences, Human Research Ethics Committee (HREC 154_2019). The study was carried out in accordance with the declaration of Helsinki involving human participants [32] and the recommended Covid precautions.

Children/adolescents aged 8–15 years admitted to either of the acute inpatient hospital settings were recruited during an onsite visit. The parent was consulted telephonically or in person for consent and study-related socio-demographic information for their child. The children/adolescents were asked to self-complete the EQ-5D-Y-5L, PedsQL, SRH and EQ-5D-Y-3L in that order. The EQ-5D-Y-5L was presented first as Janssen et al. (2008) found in the presentation of the adult measures that if the three-level version was presented first the additional levels on the five level were not considered [33]. The two versions were further separated by the PedsQL and SRH to reduce bias. The participants who returned the research packs at baseline were invited to complete a second and third measure of the EQ-5D-Y-5L, SRH and EQ-5D-Y-3L 24 and 48 h after baseline data collection to determine responsiveness.

### Data management and analysis

The sample size was powered to detect a difference in proportions between two time points in the EQ-5D-Y-3L and EQ-5D-Y-5L. It was anticipated that the effect size between time periods would be small, i.e. 0.4. A minimum total sample of 66 children was required to complete the measure at each time point to ensure a power of 90% with a significance level of 0.05.

### General performance and feasibility

The EQ-5D-Y responses and descriptive data were summarised in terms of frequency of responses. The feasibility was assessed by comparing the number of missing values for the two EQ-5D-Y measures. The ceiling of the EQ-5D-Y was defined as the proportion of children/adolescents scoring no problems in a dimension or across all five dimensions (11111). The floor effect is the proportion of children/adolescents scoring the most severe problems for a dimension or across all five dimensions (55555/33333). The absolute reduction in proportion scoring no problems or the most severe problems from the 3L to the 5L was calculated and due to the small number of respondents with an acute or chronic health condition reporting 11111 and 55555/33333 a percentage reduction was also calculated as (ceiling _EQ-5D-Y-3L_- ceiling _EQ-5D-Y-5L_)/ceiling _EQ-5D-Y-3L_.

### Redistribution properties of the EQ-5D-Y-3L to the EQ-5D-Y-5L

Paired dimension responses on the EQ-5D-Y-3L and EQ-5D-Y-5L were assessed for inconsistency using criteria established in previous studies comparing the adult EQ-5D versions [33, 34]. A response pair was considered inconsistent if the EQ-5D-Y-5L response was at least two levels away from the EQ-5D-Y-3L response. To note the youth version differed from the adult version in that level 3 on the EQ-5D-Y-3L is semantically equivalent to level 4 on the EQ-5D-Y-5L, and not level 5, thus the redistribution of level 3 (EQ-5D-Y-3L) was considered to redistribute to level 3, 4 or 5 on the EQ-5D-Y-5L. One expected that a lot of problems on the EQ-5D-Y-3L (level 3 EQ-5D-Y-3L), would redistribute to some problems (level 3 EQ-5D-Y-5L), a lot of problems (level 4 EQ-5D-Y-5L), or cannot (level 5 EQ-5D-Y-5L) on the EQ-5D-Y-5L. Similarly some problems (level 2 EQ-5D-Y-3L) would redistribute to a little bit of problems (level 2 EQ-5D-Y-5L), some problems (level 3 EQ-5D-Y-5L), or a lot of problems (level 4 EQ-5D-Y-5L) and no problems on (level 1 EQ-5D-Y-3L) would redistribute to no problems (level 1 EQ-5D-Y-5L), or a little bit of problems (level 2 EQ-5D-Y-5L). The proportion of EQ-5D-Y-3L and EQ-5D-Y-5L dimension response pairs were calculated for comparison.

### Discriminatory power

The Shannon Index (H′) and the Shannon Evenness Index (J′) were used to evaluate the discriminatory power of the EQ-5D-Y-3L and EQ-5D-Y-5L dimensions in terms of absolute and relative informativity [33, 35]. The Shannon H′ and J′ indices are defined as follows:

$$H^{\prime } = \mathop \sum \limits_{i = 1}^{L} p_{i} \,log_{2} \,p_{i} \quad {\text{and}}\quad J^{\prime } = \frac{H^{\prime}}{{H^{\prime}_{max} }}$$

where H′ is the absolute amount of informativity, L is the number of dimensions levels and p_*i*_ is the proportion of observations in the in the *i*th level where Y-3L has three levels and Y-5L has five levels. A higher H′ index reflects that the descriptive system has captured more information, the maximum H’index is 1.58 and 2.32 on the EQ-5D-Y-3L and EQ-5D-Y-5L respectively. The Shannon Evenness index (J′) reflects the spread of the responses across levels regardless of the number of levels included in the descriptive system.

### Concurrent validity

The concurrent validity of the dimension scores of the EQ-5D-Y-3L and EQ-5D-Y-5L were compared to the similar individual PedsQL items and sub-scale scores using Spearman correlations (r_s_). PedsQL summary and total scores were compared to EQ-5D-Y VAS and LSS and SRH scores with the Pearson correlation co-efficient. Correlation coefficients were interpreted according to Cohen: 0.1–0.29 low association, 0.3–0.49 moderate association and ≥ 0.5 high association [36].

### Responsiveness

Frequency and proportion of problems across the EQ-5D-Y-3L and EQ-5D-Y-5L dimensions were presented at baseline measurement (0), 24 h and 48 h later. Reporting across dimensions was dichotomised into reporting of no problems and reporting of any problems (level 2/3/4/5) to calculate absolute reduction in reporting of any problems across time (0–24 h, 24–48 h, and 0–48 h). Mean LSS and VAS scores were reported at each time point and similarly compared across time with paired t-test.

The EQ-5D-Y-3L and EQ-5D-Y-5L dimension LSS scores were presented as mean and standard deviation (SD) at each time point and the mean difference between time points (0–24 h, 24–48 h, and 0–48 h) was analysed with paired t-test and Cohen’s d effect size and the 95% confidence interval (CI). This analysis was done for the total sample as well as those who reported no change, improvement, or worsening health on the self-rated health question. Effect size was interpreted according to Cohen with 0.20, 0.50 and 0.80 indicating small, medium, and large effect sizes respectively.

All data analyses were conducted using SPSS Windows 27.0 (IBM SPSS Inc., Chicago, IL, USA) and Statistica Windows Version 13.0 (TIBCO Software Inc., Palo Alto, CA, USA).

## Results

A total of 92 children/adolescents needing acute orthopaedic management were eligible for recruitment, nine caregivers were uncontactable to obtain informed consent. A total of 83 children/participants were enrolled and completed baseline data. Seventy-eight completed the measures at 24 h and 71 at 48 h, the other participants were discharged before completion of repeat measures.

The mean age of the children/adolescents across the age groups was 11.5 years (SD 1.9). Sex of participants was similarly distributed with 47% males. Majority of the children/adolescents required surgical management for correction of congenital or acquired lower limb orthopaedic conditions (61%) including but not limited to Blount’s disease, Cerebral Palsy, Spina Bifida, Club Foot and septic or psoriatic arthritis (Table 1). The minority (29%) were admitted for surgical or conservative management of Traumatic lower limb fractures, amputations, or surgical correction of an upper limb fracture.

**Table 1:** Descriptive statistics of the sample

Traumatic orthopaedic conditions	N = 24	
	n	%
Traumatic lower limb fracture	17	71
Traumatic amputation	4	17
Traumatic upper limb fracture	3	13

Congenital or acquired lower limb orthopaedic conditions	N = 51	
Femoral osteotomy (± de-rotation)	13	21
Blount’s correction	10	16
Other*	8	13
Correction of foot deformity	5	8
Osteitis/osteomyelitis	5	8
Tendon release/lengthening	4	7
Lower limb joint epiphysiodesis	3	5
Septic/psoriatic arthritis	3	5

N = 83. *Other: Spinal surgery, arthrotomy, traction and Botox

### Feasibility

There were no missing responses across the EQ-5D-Y-5L or EQ-5D-Y-3L. The proportion of participants reporting a ceiling effect with no problems in each dimension (11111) showed a 20% relative reduction from the EQ-5D-Y-3L to the EQ-5D-Y-5L (Table 2). The relative reduction for dimensions was high and ranged from 36% (*Mobility*) to 0% (*doing Usual activities).* Only two children/adolescents reported the most severe health state (33333/55555) for the EQ-5D-Y-3L and this reduced to one for the EQ-5D-Y-5L. To note the floor of the EQ-5D-Y-3L has a label of ‘a lot of problems’ which is equivalent to level 4 on the EQ-5D-Y-5L as level 5 refers to ‘cannot’ or ‘extreme’. The number of children/adolescents reporting the most severe problems across dimensions was high for the dimensions of *doing Usual Activities* and *having Pain or Discomfort* across both measures*.* The reduction of reporting the most severe state was low with the largest reduction shown for *having Pain or Discomfort* (63%). For the dimension of *Mobility* there was an increase in reporting of the most severe problem on the EQ-5D-Y-5L compared to the EQ-5D-Y-3L. There was no significant difference in the proportion of ceiling or floor effects between the EQ-5D-Y-3L and EQ-5D-Y-5L.

**Table 2:** Ceiling and floor effect for the EQ-5D-Y-3L and EQ-5D-Y-5L

	EQ-5D-Y-3L		EQ-5D-Y-5L		Chi-square (p value)	Absolute reduction (%)	Relative reduction (%)
	n	%	n	%			
*Ceiling effect*							
11111	5	6	4	5	0.03 (p = 1.00)	1	20
MOB	14	17	9	11	1.26 (p = 0.37)	6	36
LAM	51	61	49	59	0.10 (p = 0.88)	2	4
UA	12	14	12	14	0.00 (p = 1.00)	0	0
P/D	41	49	36	43	0.61 (p = 0.54)	6	12
WSU	49	59	46	55	0.22 (p = 0.75)	4	6
*Floor effect*							
55555/33333	2	2	1	1	0.05 (p = 1.00)	1	50
MOB	48	58	50	60	0.10 (p = 0.88)	− 2	− 4
LAM	11	13	11	13	0.00 (p = 1.00)	0	0
UA	48	58	48	58	0.00 (p = 1.00)	0	0
P/D	8	10	3	4	2.43 (p = 0.21)	6	63
WSU	7	8	2	2	0.13 (p = 0.17)	6	71

N = 83, MOB, Mobility; LAM, Looking After Myself; UA, doing Usual Activities; P/D, having Pain or Discomfort; WSU, feeling Worried, Sad or Unhappy

### Redistribution properties of the EQ-5D-Y-3L to the EQ-5D-Y-5L

The dimension of *doing Usual Activities* had many inconsistencies *(25%)* which can be attributed to reporting some problems on the EQ-5D-Y-3L and cannot on the EQ-5D-Y-5L (Table 3). The inconsistency across the other dimensions was more similar and ranged from 12 to 17% *(having Pain or Discomfort to Looking After Myself).* For dimensions of *Mobility* this is largely (12%) due to moving from some problems on the EQ-5D-Y-3L to cannot on the EQ-5D-Y-5L. For the dimension of *Looking After Myself* and *feeling Worried, Sad and Unhappy* this is largely attributed to moving from some problems on the EQ-5D-Y-3L to no problems on the EQ-5D-Y-5L (17% and 8% respectively). Most respondents remain at the ceiling “no problem” (*Looking After Myself, having Pain or Discomfort* and *feeling Worried Sad or Unhappy)* or at the floor “a lot” and “cannot or extreme” (*Mobility* and *doing Usual Activities*) for both the EQ-5D-Y-3L and EQ-5D-Y-5L.

**Table 3:** Cross tabulation for the EQ-5D-Y-3L and EQ-5D-Y-5L dimension scores

EQ-5D-Y-3L	EQ-5D-Y-5L					Total inconsistent responses n (%)
**MOB**	no	a little bit	some	a lot	cannot	
No	7	5	***1***	***0***	***1***	10 (12%)
Some	***1***	12	2	1	***5***	
A lot	***1***	***1***	1	1	44	
**LAM**	no	a little bit	some	a lot	cannot	
No	43	5	***2***	***0***	***1***	14 (17%)
Some	***6***	8	3	1	***3***	
A lot	***0***	***2***	0	2	7	
**UA**	no	a little bit	some	a lot of	cannot	
No	8	0	***1***	***2***	***1***	21 (25%)
Some	***3***	4	3	3	***10***	
A lot	***1***	***3***	1	6	37	
**P/D**	no	a little bit	some	a lot	extreme	
No	33	7	***1***	***0***	***0***	7 (8%)
Some	***2***	17	10	3	***2***	
A lot	***1***	***1***	2	3	1	
**WSU**	no	a little bit	quite	really	extreme	
Not	39	8	***1***	***0***	***1***	10 (12%)
A bit	***7***	15	4	1	***0***	
Very	***0***	***1***	1	4	1	

N = 83, bold and italicised indicates inconsistent responses

MOB, Mobility; LAM, Looking After Myself; UA, doing Usual Activities; P/D, having Pain or Discomfort; WSU, feeling Worried, Sad or Unhappy

### Discriminatory power

Informativity of dimensions improves across all dimensions on the EQ-5D-Y-5L compared to the EQ-5D-Y-3L with an average improved of 0.267 with similar evenness (Table 4). *Having Pain or Discomfort* and *doing Usual Activities* showed the greatest difference in spread of information between the EQ-5D-Y-3L and EQ-5D-Y-5L.

**Table 4:** Shannon Index (H’) and Shannon Evenness Index (J’) for the EQ-5D-Y-3L and EQ-5D-Y-5L dimensions

	EQ-5D-Y-5L		EQ-5D-Y-3L		Difference in H'	Difference in J'
	H'	J'	H'	J'		
MOB	1.114	0.164	0.965	0.163	0.149	0.001
LAM	1.177	0.233	0.915	0.245	0.262	− 0.012
UA	1.242	0.169	0.952	0.165	0.290	0.004
P/D	1.324	0.223	0.939	0.283	0.385	− 0.060
WSU	1.135	0.150	0.885	0.123	0.250	0.027
average difference	0.267	− 0.008				

N = 83, MOB, Mobility; LAM, Looking After Myself; UA, doing Usual Activities; P/D, having Pain or Discomfort; WSU, feeling Worried, Sad or Unhappy

### Concurrent validity

There were missing responses from six respondents on the PedsQL scale, one of which did not complete any of the PedsQL items and were excluded from analysis. The missing item responses ranged from 1–3 across items. It was anticipated that items with similar constructs, would have a moderate to high correlation of > 0.30 [37]. Due to the difference in descriptive systems between the EQ-5D-Y and PedsQL items that were hypothesised to have a moderate to high correlation are shaded. Table 5 shows that the EQ-5D-Y-5L and EQ-5D-Y-3L had similar low to moderate association with similar items on the PedsQL generic measure except for *feeling Worried, Sad or Unhappy* and *doing Usual Activities*. Neither the EQ-5D-Y-5L nor the EQ-5D-Y-3L were associated with items of sad or worried on the PedsQL. Table 6 shows the concurrent validity of the VAS and EQ-5D-Y-5L and EQ-5D-Y-3L LSS with the PedsQL scores and Self-rated health scores. The EQ-5D-Y-5L and EQ-5D-Y-3L LSS scores showed a low association with the Physical Health PedsQL score whereas there was weak association with the PedsQL emotion sub-score and the SRH score.

**Table 5:** Spearman Correlation of EQ-5D-Y-5L and EQ-5D-Y-3L dimension scores and PedsQL item and sub-scale scores

PedsQL	EQ-5D-Y-5L					EQ-5D-Y-3L				
	MOB	LAM	UA	P/D	WSU	MOB	LAM	UA	P/D	WSU
Hard to walk; 100 m	− 0.69^**^	− 0.24^*^	− 0.35^**^	− 0.14	− 0.19	− 0.59^**^	− 0.15	− 0.25^*^	− 0.02	− 0.12
Hard to run	− 0.29^**^	0.03	− 0.31^**^	− 0.134	− 0.03	− 0.32^**^	0.06	− 0.25^*^	0.01	0.11
Hard to participate in sport / exercise	− 0.31^**^	− 0.01	*− 0.18*	− 0.08	− 0.21	− 0.28^*^	0.06	− 0.28^*^	0.05	− 0.01
Hard to lift something heavy	− 0.26^*^	− 0.15	− 0.16	− 0.10	− 0.05	− 0.22	− 0.15	− 0.20	− 0.07	0.06
Hard to bath / shower myself	− 0.16	− 0.50^**^	− 0.00	− 0.15	− 0.19	− 0.30^**^	− 0.35^**^	− 0.15	− 0.23^*^	− 0.13
Hard to do household chores	− 0.23^*^	− 0.41^**^	− 0.06	− 0.21	− 0.11	− 0.17	− 0.38^**^	− 0.02	− 0.16	0.10
Pain or aches	− 0.04	− 0.11	0.09	− 0.53^**^	− 0.09	− 0.13	− 0.21	0.22	− 0.44^**^	− 0.04
Low energy levels	− 0.07	− 0.20	0.05	− 0.15	− 0.05	− 0.16	− 0.18	− 0.17	− 0.26^*^	0.02
Physical Health Summary Score	− 0.45^**^	− 0.36^**^	− 0.18	− 0.26^*^	− 0.17	− 0.47^**^	− 0.28^*^	− 0.21	− 0.20	0.02
Afraid or scared	− 0.27^*^	− 0.15	0.06	0.04	− 0.23^*^	− 0.24^*^	− 0.11	0.02	0.01	− 0.17
Sad	0.013	0.05	0.17	0.11	*− 0.12*	− 0.03	0.09	0.12	0.10	*− 0.13*
Angry	− 0.00	− 0.06	0.21	0.06	0.08	0.00	− 0.03	0.17	0.05	− 0.12
Trouble sleeping	− 0.06	− 0.03	0.05	− 0.22	− 0.06	− 0.18	− 0.03	0.07	− 0.21	− 0.08
Worry about what will happen to me	− 0.16	− 0.02	− 0.05	0.02	*− 0.04*	− 0.12	0.09	− 0.06	− 0.10	*− 0.19*
Emotional sub-score	− 0.16	− 0.06	0.14	− 0.06	*− 0.12*	− 0.22^*^	− 0.03	0.06	− 0.11	*− 0.21*
Trouble getting along with other kids/teenagers	− 0.00	0.06	− 0.06	0.09	− 0.02	0.13	0.03	0.06	0.14	0.12
Other kids/teenagers do not what to be my friend	− 0.02	− 0.05	− 0.07	0.02	− 0.10	0.09	− 0.05	− 0.05	0.01	− 0.03
Other kids/teenagers tease me	− 0.22	− 0.35^**^	− 0.09	− 0.29^**^	− 0.15	− 0.14	− 0.33^**^	− 0.17	− 0.30^**^	− 0.19
Cannot do things others my age can do	− 0.03	− 0.04	*0.05*	0.16	− 0.11	0.00	0.03	*− 0.08*	0.13	0.06
Hard to keep up with others	− 0.02	0.08	− 0.02	0.23^*^	− 0.08	− 0.01	0.11	− 0.06	0.32^**^	0.12
Social sub-score	− 0.09	− 0.10	− 0.05	0.10	− 0.15	0.00	− 0.08	− 0.08	0.13	0.05
Hard to pay attention in class	0.09	− 0.01	0.12	0.03	− 0.05	0.05	0.02	0.02	− 0.09	− 0.05
Forget things	− 0.17	− 0.02	− 0.01	0.02	− 0.08	− 0.24^*^	− 0.08	− 0.13	− 0.03	− 0.06
Trouble keeping up with my schoolwork	− 0.05	− 0.09	− 0.01	0.00	− 0.11	− 0.18	− 0.11	− 0.17	0.06	− 0.11
Miss school because of not feeling well	0.11	0.03	0.02	0.10	− 0.19	0.08	0.00	0.04	0.08	− 0.11
Miss school to go doctor or hospital	0.093	− 0.12	0.20	0.13	− 0.01	0.03	− 0.03	0.11	0.05	0.00
School sub-score	− 0.08	− 0.08	0.04	0.10	− 0.16	− 0.14	− 0.08	− 0.09	0.06	− 0.16

N = 82, * p < 0.05 and **p < 0.001 (2-tailed). Cells in bolditalic and italic were hypothesised to have a moderate to high correlation are shaded. Bolded correlations have a moderate association > 0.30, correlations are negative as a higher PedsQL score indicates a better HRQoL. MOB, Mobility; LAM, Looking After Myself; UA, doing Usual Activities; P/D, having Pain or Discomfort; WSU, feeling Worried, Sad or Unhappy

**Table 6:** Summary of concurrent validity with the PedsQL summary and sub-scores, Self-rated health and EQ-5D-Y VAS scores with Pearson Correlation

	PedsQL						Self-rated Health
	Total	Summary Scores					
		Physical	Psychosocial	Psychosocial sub-scores			
				Emotional	Social	School	
VAS	− 0.24^*^	− 0.42^**^	− 0.09	− 0.10	− 0.10	0.00	− 0.27^*^
EQ-5D-Y-5L LSS	0.20	0.25^*^	0.13	0.22^*^	− 0.07	0.10	0.31^**^
EQ-5D-Y-3L LSS	0.20	0.25^*^	0.13	0.22^*^	− 0.03	0.07	0.40**

N = 82, *p < 0.05 and **p < 0.001 level (2-tailed). Cells shaded in grey have a moderate association > 0.30, A higher LSS indicated a worse health state. VAS, Visual Analogue Scale; LSS, Level Sum Score; GP, General Population

### Responsiveness

Table 7 shows the absolute difference in the reporting of “any problems” was generally higher on the EQ-5D-Y-5L than the EQ-5D-Y-3L across dimensions. The largest difference was seen between baseline and 48 h on both measures. The difference in reporting any problems was smaller for 24–48 h compared to 0–24 h on the EQ-5D-Y-5L andEQ-5D-Y-3L, with the exception of *having Pain or Discomfort* on the EQ-5D-Y-3L.. The greatest change in reporting of problems was seen for *Mobility* across both measures. The EQ-5D-Y-5L and EQ-5D-Y-3L had similar performance with change over the three time periods with an increase in reporting of no problems and decrease in reporting of problems on individual levels. The VAS score showed significant differences between 0–24 h and 0–48 h.

**Table 7:** EQ-5D-Y-5L and EQ-5D-Y-3L scores across three days and the difference in percentage of reporting problems between days

	EQ-5D-Y-5L									EQ-5D-Y-3L								
	Baseline		24 h		48 h		Δ reporting problems			Baseline		24 h		48 h		Δ reporting problems		
	(n = 83)		(n = 78)		(n = 71)		0–24 h	24–48 h	0–48 h	(n = 83)		(n = 78)		(n = 71)		0–24 h	24–48 h	0–48 h
	n	%	n	%	n	%	%	%	%	n	%	n	%	n	%	%	%	%
*MOB*																		
No	9	11	16	21	22	31	91	48	182	14	17	20	26	24	34	53	31	100
Any problem	74	89	61	78	49	69	− 12	− 12	− 22	69	83	57	73	47	66	− 12	− 10	− 20
*LAM*																		
No	49	59	53	68	52	73	15	7	24	51	61	58	74	54	76	21	3	25
Any problem	34	41	24	31	19	27	− 24	− 13	− 34	32	39	19	24	17	24	− 38	0	− 38
*UA*																		
No	12	14	12	15	12	17	7	13	21	12	14	11	14	14	20	0	43	43
Any problem	71	86	65	83	59	83	− 3	0	− 3	71	86	66	85	57	80	− 1	− 6	− 7
*P/D*																		
No	36	43	42	54	43	61	26	13	42	41	49	44	56	49	69	14	23	41
Any problem	47	57	35	45	28	39	− 21	− 13	− 32	42	51	33	42	22	31	− 18	− 26	− 39
*WSU*																		
Not	46	55	52	67	53	75	22	12	36	49	59	57	73	54	76	24	4	29
Any problem	37	45	25	32	18	25	− 29	− 22	− 44	34	41	20	26	17	24	− 37	− 8	− 41
*VAS*																		
Mean (SD)	66.3 (26.3)		76 (26)		81 (25)		p < 0.001	p = − 0.077	p < 0.001									

MOB, Mobility; LAM, Looking After Myself; UA, doing Usual Activities; P/D, having Pain or Discomfort; WSU, feeling Worried, Sad or Unhappy, VAS, Visual Analogue Scale

Paired differences between time periods (0–24, 24–48 and 0–48 h) are significant for the total sample on the EQ-5D-Y-5L LSS and all time periods except for 24–48 h for the EQ-5D-Y-3L LSS (Table 8). For those respondents who reported an improvement in their SRH the EQ-5D-Y-5L and EQ-5D-Y-3L LSS showed significant paired differences with moderate to high effect sizes which were greater for the EQ-5D-Y-5L than EQ-5D-Y-3L. For those who reported no change in SRH the EQ-5D-Y-5L and EQ-5D-Y-3L similarly recorded some differences with small to medium effect size. The difference in EQ-5D-Y-5L nor EQ-5D-Y-3L were significantly different for those who reported worsened health over time.

**Table 8:** Paired differences of EQ-5D-Y-3L and EQ-5D-Y-5L Level Sum Score (LSS) at baseline, 24 h and 48 h

	Baseline LSS	24 h LSS	Paired differences		
	Mean (SD)	Mean (SD)	Mean (SD)	p	Effect Size (95% CI)
*All (n* = *77)*					
EQ-5D-Y-5L	13.64 (3.93)	12.36 (3.89)	1.27 (3.01)	< 0.001	0.43 (0.19, 0.66)
EQ-5D-Y-3L	9.55 (2.21)	8.77 (2.05)	0.78 (1.93)	< 0.001	0.40 (0.17, 0.64)
*No change (n* = *43)*					
EQ-5D-Y-5L	13.56 (3.57)	12.79 (3.18)	0.67 (2.83)	0.126	0.24 (− 0.07, 0.54)
EQ-5D-Y-3L	9.42 (2.28)	8.84 (1.80)	0.58 (1.78)	0.038	0.33 (0.02, 0.63)
Improve (n = 22)					
EQ-5D-Y-5L	13.82 (4.54)	11.09 (4.42)	2.73 (2.91)	< 0.001	0.94 (0.42, 1.43)
EQ-5D-Y-3L	10.00 (1.98)	8.45 (2.37)	1.55 (2.30)	0.005	0.67 (0.20, 1.13)
*Worsen (n* = *12)*					
EQ-5D-Y-5L	13.58 (4.32)	12.83 (4.59)	0.75 (3.11)	0.421	0.24 (− 0.34, 0.81)
EQ-5D-Y-3L	9.17 (2.44)	9.08 (2.39)	0.08 (1.31)	0.830	0.06 (− 0.50, 0.63)

	24 h LSS	48 h LSS			
*All (n* = *71)*					
EQ-5D-Y-5L	12.38 (3.79)	11.51 (3.76)	0.87 (3.15)	0.022	0.28 (0.04, 0.51)
EQ-5D-Y-3L	8.82 (2.00)	8.51 (1.97)	0.31 (1.70)	0.128	0.18 (− 0.05, 0.41)
*No change (n* = *42)*					
EQ-5D-Y-5L	13.62 (4.22)	12.55 (3.83,0.59)	1.07 (3.10)	0.031	0.35 (0.03,0.66)
EQ-5D-Y-3L	9.50 (2.17)	8.90 (1.97)	0.60 (1.98)	0.058	0.30 (− 0.01,0.61)
*Improve (n* = *20)*					
EQ-5D-Y-5L	13.30 (4.18)	12.20 (3.74)	1.10 (2.92)	0.108	0.38 (− 0.08, 0.82)
EQ-5D-Y-3L	9.20 (2.44)	8.60 (2.06)	0.60 (1.76)	0.144	0.34 (− 0.11, 0.79)
*Worsen (n* = *9)*					
EQ-5D-Y-5L	12.00 (4.12)	13.22 (4.49)	− 1.22 (3.70)	0.351	− 0.33 (− 0.99,0.35)
EQ-5D-Y-3L	8.89 (2.09)	9.22 (1.92)	− 0.33 (1.12)	0.397	− 0.30 (− 0.96,0.38)

	Baseline LSS	48 h LSS			
*All (n* = *71)*					
EQ-5D-Y-5L	13.56 (4.01)	8.51 (1.97)	5.06 (3.45)	< 0.001	1.46 (1.12, 1.79)
EQ-5D-Y-3L	9.58 (2.19)	8.51 (1.97)	1.07 (2.08)	< 0.001	0.52 (0.26, 0.76)
*No change (n* = *32)*					
EQ-5D-Y-5L	13.66 (3.21)	11.84 (3.49)	1.81 (3.41)	0.005	0.53 (0.16, 0.90)
EQ-5D-Y-3L	9.69 (1.75)	8.81 (1.96)	0.88 (2.11)	0.025	0.42 (0.05, 0.77)
*Improve (n* = *29)*					
EQ-5D-Y-5L	13.69 (4.89)	11.34 (3.88)	2.35 (3.78)	0.002	0.62 (0.22,1.01)
EQ-5D-Y-3L	9.55 (2.47)	8.17 (1.98)	1.38 (2.13)	0.002	0.65 (0.24, 1.05)
*Worsen (n* = *10)*					
EQ-5D-Y-5L	12.90 (3.81)	10.9 (4.53)	2.00 (3.68)	0.120	0.54 (− 0.13, 1.20)
EQ-5D-Y-3L	9.30 (2.75)	8.50 (2.01)	0.80 (1.93)	0.223	0.41 (− 0.24, 1.05)

Calculated using EQ-5D-Y-3L/ EQ-5D-5L level sum scores (LSS), a higher score indicates a worse health state, LSS, level sum score; SD, standard deviation; CI, Confidence Interval

When comparing those with unchanged and improved SRH those with improved health had a significantly larger LSS (better health state) on both the EQ-5D-Y-5L and EQ-5D-Y-3L LSS between baseline and 24 h (Table 9). The health state was greater for those with improved health across the other time points too except for the EQ-5D-Y-5L at 24–48 h. For those with unchanged versus worsened the health state was better for EQ-5D-Y-5L at 0–24 and 0–48 h but significantly worse at 24–48 h. For the EQ-5D-Y-3L LSS the health state was worse than those with worse SRH score at 0–24 h, 24–48 h but better at 0–48 h. Those with improved SRH had a slightly higher LSS (worse SRH) than those with worsened SRH on the EQ-5D-Y-5L and EQ-5D-Y-3L at all three time points.

**Table 9:** Change in EQ-5D-Y-5L and EQ-5D-Y-3L level sum scores (LSS) over time and between unchanged, improve or worsened self-rated health

	Change in EQ-5D-Y-5L LSS			Change in EQ-5D-Y-3L LSS		
	Mean difference (95% CI)	p value	Effect size	Mean difference (95% CI)	p value	Effect size
*Baseline—24 h*						
Unchanged vs Improved	− 2.05 (− 3.55, − 0.55)	0.008	0.72 (0.19,1.24)	− 0.96 (1.99,0.07)	0.066	0.49 (− 0.33,0.10)
Unchanged vs worsened	− 0.76 (− 1.97, 1.82)	0.937	− 0.26 (− 0.66, 0.61)	0.50 (− 0.61,1.60)	0.371	0.30 (− 0.35, 0.94)
Improve vs worsened	1.98 (− 0.20, 4.16)	0.074	0.66 (− 0.64, 1.38)	1.46 (− 0.01, 2.94)	0.052	0.72 (− 0.01, 1.44)
*24–48 h*						
Unchanged vs Improved	0.41 (− 1.59, 1.67)	0.961	0.01 (− 0.52, 0.55)	− 0.14 (− 1.1, 0.82)	0.767	− 0.08 (− 0.61, 0.45)
Unchanged vs worsened	2.41 (0.30, 4.53)	0.026	0.84 (0.99, 1.58)	0.69 (− 0.50, 1.88)	0.025	0.43 (− 0.30, 1.15)
Improve vs worsened	2.37 (− 0.61, 5.36)	0.115	0.65 (− 0.16, 1.45)	0.833 (− 0.33, 1.00)	0.235	0.49 (− 0.31, 1.28)
*Baseline—48 h*						
Unchanged vs Improved	− 0.67 (− 2.52, 1.19)	0.475	− 0.18 (− 0.68, 0.32)	− 0.52 (− 1.61, 0.57)	0.343	− 0.24 (− 0.75, 0.26)
Unchanged vs worsened	− 0.44 (− 2.56, 1.69)	0.681	− 0.12 (− 0.69, 0.45)	− 0.64 (− 1.84, 0.56)	0.288	− 0.31 (− 0.89, 0.26)
Improve vs worsened	0.34 (− 2.45, 3.14)	0.804	0.09 (− 0.63, 0.81)	0.58 (− 0.97, 2.13)	0.453	0.28 (− 0.45,0.10)

Calculated using EQ-5D-Y-3L/5L level sum scores (LSS), a higher score indicates a worse health state

## Discussion

The aim of this study was to investigate the feasibility (missing responses), redistribution and discriminatory power of dimension responses, concurrent validity, and responsiveness of the EQ-5D-Y-3L and the EQ-5D-Y-5L. Children/adolescents receiving acute orthopaedic management were considered a suitable population for comparison of the EQ-5D-Y-3L and EQ-5D-Y-5L as they were likely to report problems across all dimensions and show improvement from baseline testing to 48 h after which they were likely to be discharged. Furthermore, the adult measure was shown to have good psychometric properties in orthopaedic patient groups [38–40].

The distribution of scores across levels was greater in this sample with more acute health conditions for the EQ-5D-Y-5L than the EQ-5D-Y-3L as evident with the reporting of the Shannon J’ index [7, 14, 15]. As such the reporting of ceiling (11111) and floor (33333/55555) were very low (< 6%) across the EQ-5D-Y-5L and EQ-5D-Y-3L with no significant differences between versions. At an individual dimension level there was however a decrease in reporting of no problems on the EQ-5D-Y-5L. As anticipated, this was most notable for the dimension of mobility (36% relative reduction) with most children experiencing management for lower limb orthopaedic conditions. Similarly high relative reduction in reporting of most severe problems in *having Pain or Discomfort* (63%) and *feeling Worried, Sad or Unhappy* (71%) was observed. Surprisingly the reporting of most severe problems in *Mobility* increased for the EQ-5D-Y-5L compared to the EQ-5D-Y-3L and could possibly be due to the more definitive wording of ‘cannot’ on the EQ-5D-Y-5L compared to ‘a lot’ on the EQ-5D-Y-3L. This was similarly observed with the high redistribution of scores across the other five levels with many respondents moving from ‘some problems’ to ‘cannot’ for dimensions of *doing Usual Activities* and *Mobility.*

Although it is unclear why respondents gave inconsistent responses for the dimension of *Usual Activities* it may be attributed to consideration of different examples given for this dimension, these may have further been influenced by answering the PedsQL between the EQ-5D-Y-5L and EQ-5D-Y-3L. Moving from ‘some problem’ on the EQ-5D-Y-3L to ‘no problems’ on the EQ-5D-Y-5L for dimensions of *feeling Worried, Sad or Unhappy* and *Looking After Myself* is not as clear but may be due to an order effect with answering the EQ-5D-Y-3L after the EQ-5D-Y-5L, PedsQL and SRH. The reduction in ceiling effect was not significant in moving from the EQ-5D-Y-3L to the EQ-5D-Y-5L the increased use of levels on the EQ-5D-Y-5L improved its discriminatory power with increased complexity (as suggested by the inconsistent responses). Furthermore, moving from level 3 (a lot) on the EQ-5D-Y-3L to level 5 (cannot), rather than the semantically equivalent level 4, on the EQ-5D-Y-5L indicates that the new level “cannot” on the EQ-5D-Y-5L is needed. This difference in wording of the most severe level between the EQ-5D-Y-3L and EQ-5D-Y-5L further poses a challenge for the interpretation of inconsistency as the level 3 on the EQ-5D-Y-3L semantically maps to level 3, 4 or 5 on the EQ-5D-Y-5L. On assessment of the adult versions mapping from level 3 on the EQ-5D-3L would be inconsistent if mapped to level 3 on the EQ-5D-5L, this would increase the inconsistencies between the two youth versions.

The discriminatory power of the EQ-5D-Y-5L showed a large improvement with the expanded levels of the EQ-5D-Y-5L compared to the EQ-5D-Y-3L (average H′ = 0.267). This was larger than the average difference reported by Verstraete et al. [7] in those receiving acute/chronic medical care and the general population (average H′ = 0.094) or by Wong et al. [14, 15] for those with idiopathic scoliosis (average H′ = 0.024). The evenness of the distribution of responses on the EQ-5D-Y-5L was retained with a low difference in index scores (J′ = − 0.008).

Marson et al. [16] recommended that EQ-5D-Y or the PedsQL be used to measure quality of life in children with fractures, the latter due to its performance in children with cancer. The results of this study however show that there is only low to moderate correlation between EQ-5D-Y-3L and EQ-5D-Y-5L dimension scores and similar PedsQL items. The physical health items showed association as expected given the impact of orthopaedic intervention (mostly lower limb) on mobility. However, the correlations in the emotion, social and school sub-scales was poor despite reporting problems with both *doing Usual Activities* and *feeling Worried, Sad and Unhappy* on the EQ-5D-Y-3L and EQ-5D-Y-5L. This could be attributed to the fact that the recall period of ‘Today’ on the EQ-5D-Y-3L and EQ-5D-Y-5L is more appropriate for those receiving acute medical management than the longer recall period of the PedsQL [41]. This could further indicate the complementary item structure of the EQ-5D-Y-3L/EQ-5D-Y-5L and PedsQL.

Although the absolute difference in reporting of problems across dimensions is not able to discriminate between the magnitude and/or direction of change in the EQ-5D-Y-5L and EQ-5D-Y-3L dimensions it gives an indication of change for comparison between the measures. As such the EQ-5D-Y-5L and EQ-5D-Y-3L had similar performance of change over time with increase in reporting of no problems and decrease in report of problems across time periods, most notably for the longest time period of 0–48 h. The EQ-5D-Y-5L generally showed greater change than the EQ-5D-Y-3L across all dimensions. As anticipated with acute surgical or medical intervention the greatest change in problems was seen on the EQ-5D-Y-5L and EQ-5D-Y-3L for dimension of *Mobility,* [38]. This change was supported with a paired difference of the LSS across time periods which similarly showed greater differences between 0–24 h and 0–48 h. The difference was significant and large for those who had improved SRH between 0–24 h and 0–48 h. Despite reporting no change in SRH there was a significant, medium improvement in LSS for the EQ-5D-Y-5L and EQ-5D-Y-3L for those with unchanged SRH. This could be due to the fact that the SRH question used was not sensitive enough to change in health state or that the LSS overestimated this improvement for the group who reported unchanged SRH. As anticipated no significant change was detected for those with worsened self-reported health.

When comparing those with unchanged and improved SRH those with improved health showed a significantly better health state on the EQ-5D-Y-5L and EQ-5D-Y-3L LSS between 0 and 24 h. It was expected that those who reported worse SRH score would have a higher LSS (worse health state) than those who had a SRH showing improvement [15]. Counterintuitively those with improved SRH had a slightly higher LSS (worse SRH) than those with worsened SRH on the EQ-5D-Y-5L and EQ-5D-Y-3L at all three time points. This could be that the SRH was not sensitive to interpret the change over time and/or that the health of the children fluctuated too much during the acute period [42].

### Study limitations

The assessment of feasibility of the EQ-5D-Y-3L and EQ-5D-Y-5L was limited to missing responses and no data was collected on completion time, qualitative assessment or participant preferences. Furthermore, the generalisability of the responsiveness results is limited as data is not collected before and after intervention [43] but rather over time in an acute facility. The heterogeneity of the orthopaedic group, including non-elective management did not allow for pre- and post- intervention data collection. The responses may have been influenced by recall bias with either the best, worst or average health state selected for the specified time period [44]. This could further have impacted on the responsiveness results if the recall was not consistently considered across repeat measures. Furthermore, a recalibration response shift may have further biased the results where the respondent’s point of view has changed [44].

## Conclusion

The English version of the EQ-5D-Y-5L is a valid and responsive extension of the EQ-5D-Y-3L for children/adolescents receiving acute orthopaedic intervention. The expanded levels reduce the ceiling effect and floor effect on the EQ-5D-Y-3L, most notably ceiling effect was reduced, although not significantly, for dimensions of *Mobility* and floor effect in dimensions of *having Pain or Discomfort* and *feeling Worried, Sad or Unhappy*. The relative informativity of report across the dimensions has increased on the EQ-5D-Y-5L compared to the EQ-5D-Y-3L with retention of the evenness of reporting. The concurrent validity of the EQ-5D-Y-5L was comparable to the EQ-5D-Y-3L. The EQ-5D-Y-5L generally showed greater change than the EQ-5D-Y-3L across all dimensions with the greatest change observed for 0–48 h. Responsiveness was comparable across the EQ-5D-Y-3L and EQ-5D-Y-5L for those with improved SRH. Greater sensitivity to change may be observed on comparison of utility weights, once preference-based value sets are available for the EQ-5D-Y-5L.

## Data Availability

The datasets used and/or analysed during the current study are available from the corresponding author on reasonable request.

## Abbreviations

LAM: Looking after myself
MOB: Mobility
P/D: Having pain or discomfort
PedsQL: Pediatric Quality of Life Inventory
SRH: Self-Rated Health
UA: Doing usual activities
WSU: Feeling worried, sad or unhappy
